# Honey Bee Proteolytic System and Behavior Parameters under the Influence of an Electric Field at 50 Hz and Variable Intensities for a Long Exposure Time

**DOI:** 10.3390/ani11030863

**Published:** 2021-03-18

**Authors:** Paweł Migdał, Agnieszka Murawska, Aneta Strachecka, Paweł Bieńkowski, Adam Roman

**Affiliations:** 1 Department of Environment, Hygiene and Animal Welfare, Wroclaw University of Environmental and Life Sciences, 25 C.K. Norwida St., 51-630 Wroclaw, Poland; agnieszka.murawska@upwr.edu.pl (A.M.); adam.roman@upwr.edu.pl (A.R.); 2 Institute of Biological Basis of Animal Production, Faculty of Biology, Animal Sciences and Bioeconomy, University of Life Sciences in Lublin, Akademicka 13, 20-950 Lublin, Poland; aneta.strachecka@up.lublin.pl; 3 Telecommunications and Teleinformatics Department, Wroclaw University of Science and Technology, 27 Wybrzeze Wyspianskiego St., 50-370 Wroclaw, Poland; pawel.bienkowski@pwr.edu.pl

**Keywords:** proteases, behavior, proteolytic enzymes, electromagnetic field, honey bee immunity

## Abstract

**Simple Summary:**

The amount of electromagnetic field (EMF) in the environment emitted by electrical and electronic devices, mobile phone masts, or power lines is constantly increasing. Honey bee can be exposed to the EMF in the environment, ^a^nd the influence of this factor on bees is still under consideration. Studying the impact of EMF on honey bees can give valuable information about whether it poses a threat to them. The honey bee is an important pollinator, playing a significant role in maintaining biodiversity and food production. Our research showed that a 50 Hz electric field at various intensities reduced the number of occurrences of walking, contacts between individuals, and self-grooming, and increased the activity of proteases, which are involved in the immune system response.

**Abstract:**

The effect of an artificial electromagnetic field on organisms is a subject of extensive public debate and growing numbers of studies. Our study aimed to show the effect of an electromagnetic field at 50 Hz and variable intensities on honey bee proteolytic systems and behavior parameters after 12 h of exposure. Newly emerged worker bees were put into cages and exposed to a 50 Hz E-field with an intensity of 5.0 kV/m, 11.5 kV/m, 23.0 kV/m, or 34.5 kV/m. After 12 h of exposure, hemolymph samples were taken for protease analysis, and the bees were recorded for behavioral analysis. Six behaviors were chosen for observation: walking, flying, self-grooming, contact between individuals, stillness, and wing movement. Bees in the control group demonstrated the highest number of all behavior occurrences, except flying, and had the lowest protease activity. Bees in the experimental groups showed a lower number of occurrences of walking, self-grooming, and contacts between individuals than the control bees and had significantly higher protease activity than the control bees (except that of alkaline proteases in the 23.0 kV/m group).

## 1. Introduction

The amount of electromagnetic field in the environment emitted by electrical and electronic devices, mobile phone masts, or power lines is constantly increasing [1]. The effect of the artificial electromagnetic field on organisms is the subject of extensive public debate and growing numbers of studies. The influence of the electromagnetic field on the honey bee has also been a topic of various research projects. The honey bee as an element of the environment is constantly exposed to various stressors, including electromagnetic fields of various frequencies and intensities.

50 Hz is a widely used power frequency in most countries [2,3]. If a honey bee flies at a height of about 2 m above ground in an open space near a power line it is exposed to an E-field with an intensity of 10–12 kV/m. If high obstacles appear in the honey bee’s way, it flies about five or more meters above the ground so it is exposed to an E-field with an intensity of 5–7 kV/m [3,4,5].

Bees have been proved to avoid feeding places exposed to a static electromagnetic field (1.5 kV/m) [6]. The success of the foraging of bees was limited by exposure to a low-frequency electromagnetic field [7]. A 60 Hz electromagnetic field > 150 kV/m caused wing, antennae, and body vibrations [8]. Migdał et al. [9] show that bees exposed to a 50 Hz electric field (E-field) at various intensities changed the activity of the bees. Bindokas et al. [10] show that the exposition of the honey bee to conductive tunnels increased mortality. Moreover, an electromagnetic field has an impact on the honey bee’s physiology by modifying pupal development (mobile phone radiation) [11], increasing oxygen consumption (static field 1.4–2.8 kV/m) [12], or changing biochemical parameters (50 Hz E-field at 5.0 kV/m, 11.5 kV/m, 23.0 kV/m, and 34.5 kV/m for 1, 3,6, and 12 h) [13,14]. Thus, previous studies indicate that an electromagnetic field may be one of the threats to honey bees.

Numerous environmental factors, including anthropogenic ones, have an influence on the activity of the honey bee’s immune system. Broadly understood environmental pollution weakens the bee’s immune system and reduces colony health [15,16]. Two types of immunity can be distinguished in honey bees: individual and social. Individual immunity includes anatomical barriers, cellular and humoral immunity, while social includes behavioral immunity [17,18].

One of the important individual barriers is proteases, which occur both inside the honey bee organism and on the surface of its body [14]. The proteolytic system in the honey bee (*Apis mellifera* L.) organism is involved in crucial processes, such as protein digestion, receptor activation, the release of hormones, and activation of the zymogens [19,20,21,22]. Among their various functions, these enzymes play a significant role in the activity of the immune system. They are one of the basic lines of defense against pathogens [23].

Behavioral immunity consists of, among others, hygienic instinct, bee fever, and absconding. Bees show grooming, which includes self-grooming, and social-grooming (allo-grooming). This behavioral complex helps to reduce, for example, *Varroa destructor* infestation [16,24].

Our study aimed to show the effect of an electromagnetic field at 50 Hz and variable intensities on honey bee proteolytic systems and behavior parameters after exposure for 12 h.

## 2. Materials and Methods

### 2.1. Bees

Queens originating from the same mother-queen colony were inseminated with the semen of drones from the same father-queen colony. Ten mother queens were randomly selected and kept in isolators with empty Dadant combs (435 × 300 mm) for egg-laying. Each queen was kept in a separate bee colony. On the 20th day of bee development, the combs with the already sealed worker bee brood were transferred to an incubator with constant conditions (temperature of 34.4 °C ± 0.5 °C and relative humidity of 70% ± 5%) for emerging without adult bees. The combs were transported at the same time and put together in one incubator. Feed (honey and bee bread) was provided ad libitum.

### 2.2. Experimental Design

One-day-old workers were randomly placed in 50 wooden cages (20 × 15 × 7 cm). Each cage contained 100 workers and two inner feeders with a 50% sucrose solution. Bees were fed ad libitum. The bees were divided into four experimental groups which were exposed to the following 50 Hz E-field intensities: 5.0 kV/m, 11.5 kV/m, 23.0 kV/m, or 34.5 kV/m for 12 h, and the control group. The control group was not treated by the artificial E-field; in this group, the bees were under the influence of an electromagnetic field < 1.00 kV/m. Each group consisted of ten cages. The group name was the E-field intensity to which the bees were exposed. The control group is marked with the letter C.

### 2.3. E-Field Generation

A homogeneous 50 Hz E-field was generated in the exposure system in the form of a plate capacitor with the distance of 20 cm between two electrodes constructed as a squared cage made out of wire mesh, as per Migdał et al. [9]. In most countries, 50 Hz is a widely used power frequency [25]. The E-field intensity and the homogeneity in the test area were verified by an LWiMP accredited testing laboratory (certification AB-361 of Polish Centre for Accreditation) using an ESM-100-m No. 972,153 with calibration certificate LWiMP/W/070/2017, dated 15/02/2017 and issued by the accredited calibration laboratory PCA AP-078. The measurements were done at points of a 10 × 10 × 5 cm^3^ mesh inside an empty emitter. The stability of the electric field was maintained by permanently monitoring the voltage applied to the exposure system using a control circuit. The field intensity was fixed at 5.0 kV/m, 11.5 kV/m, 23.0 kV/m, or 34.5 kV/m. Changes in the homogeneity and stability of the E-field intensity were no higher than ±5% in the emitter, to which the bees were exposed during the whole experiment.

### 2.4. Protease Analysis

Hemolymph samples were collected from 100 bees randomly taken from each group. The hemolymph was taken after exposure by removing the antennae of a live bee using sterile tweezers as per Migdał et al. [26]. The hemolymph sample was collected in sterile glass capillaries with a volume of 20 μL end-to-end without anticoagulant. The prepared capillaries were placed in 1.5 mL Eppendorf tubes filled with 150 μL of 0.6% NaCl. The test tubes were placed on the cooling block during this procedure. The prepared tubes were transferred to a cryo-box and then frozen at −80 °C [27]. Determinations of the acidic, neutral, and alkaline protease activities were done according to the Anson method [28] modified by Strachecka and Demetraki-Paleolog [16]. The activities of acidic proteases were assayed in a buffer of 100 mM glycine-HCl at pH 2.4, neutral ones in a buffer of 100 mM Tris-HCl at pH 7.0, and alkaline ones in a buffer of 100 mM glycine-NaOH at pH 11.2 using the method described by Łoś and Strachecka [27]. The samples of hemolymph were collected immediately after the end of exposure to the E-field.

### 2.5. Behavior Analysis

Twenty-one bees were randomly taken from each group and were placed in a behavioral assessment station made of glass, with a height of 20 cm and a diameter of 40 cm. Observations were conducted with the use of recorded material (offline). Three bees were recorded at the same time for 360 s (60 s for adaptation to location change and 300 s for analysis) using a SONY HDR-CX240E camera (Lund, Sweden). Recorded videos were transferred to a computer on which Noldus Observer XT 9.0 software was installed. Six basic behaviors were selected for observation, i.e., walking, self-grooming (self-cleaning of the body surface, cleaning of antennae, and cleaning of proboscis), flying (between the walls, the bottom and the lid of the container), stillness (time when the bee remained motionless), contact between individuals (including trophallaxis and allo-grooming), and wing movement (exposed Nasonov’s gland).

For behavioral analysis using the Noldus Observer XT 9.0 software, a project with a mutually exclusive type of behavior was used (observation for each bee was done separately and only one bee was observed at any one time). The project did not use behavior modifiers in the form of changing conditions or interfering with the insects, as all individuals were assessed under the same conditions. Independent variables in the form of age, body condition, and damage were excluded, as per Migdał et al. [9].

For analysis, we chose the average duration of behavior (how much time, on average, bees from one group spent on the behavior) and the number of individual behavior occurrences (how many times during the observation individuals from the group displayed the behavior). The recording of the bees was immediately after the end of exposure to the E-field.

### 2.6. Data Analysis

The normality of the data distribution was analyzed using the Shapiro–Wilk test. The statistical significance of data between groups was determined by the Kruskal–Wallis test and Dunn’s post hoc rank sum comparision using the package “pgirmess” for “kruscalmc” function. For all tests, RStudio [29] was used with a significance level of α = 0.05.

## 3. Results

### 3.1. Protease Analysis

**Figure 1:**
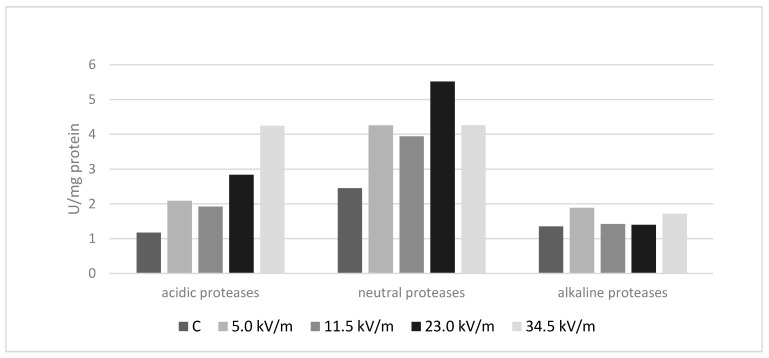
The average protease activity in bee organisms after 12 h under the influence of an E-field at 50 Hz and an intensity of 5.0 kV/m, 11.5 kV/m, 23.0 kV/m, or 34.5 kV/m. The group name is the E-field intensity to which the bees were exposed. The control group is marked with the letter C.

In all experimental groups, the level of protease activity was higher than in the control group (Figure 1). Differences between all experimental groups and the control group were statistically significant except for the 23.0 kV/m group in the case of alkaline proteases (Table 1).

An intensity of 5.0 kV/m increased the activity of acidic proteases by 78%, neutral by 74%, and alkaline by 40% compared to the control group. Bees treated with an intensity of 11.5 kV/m were characterized by 63% higher activity of acidic proteases, 61% higher neutral protease activity, and 5% higher alkaline protease activity in comparison to the control bees. An intensity of 23.0 kV/m caused an increase of acidic protease activity by 142%, neutral protease activity by 125%, and alkaline protease activity by 4% compared to the control group. Bees exposed to an E-field with an intensity of 34.5 kV/m had 261% higher acidic protease activity, 74% higher neutral protease activity, and 27% higher alkaline protease activity compared to the control bees.

### 3.2. Acidic Proteases

The highest activity of acidic proteases was recorded in bees treated with an intensity of 34.5 kV/m, while the lowest was in the control group. Among the experimental groups, the least influence on acidic protease activity was for an intensity of 11.5 kV/m (Figure 1). All differences between the groups were statistically significant (Table 1) (*p*-value < 2.2 × 10^−16^).

### 3.3. Neutral Proteases

An intensity of 23.0 kV/m caused the highest increase of neutral protease activity. Control bees were characterized by the lowest activity of neutral proteases. Among the experimental groups, the least influence on neutral protease activity was for an intensity of 11.5 kV/m (Figure 1). The activity of neutral proteases in bees from the 5.0 kV/m and 34.5 kV m groups did not differ significantly (Table 1) (*p*-value < 2.2 × 10^−16^).

### 3.4. Alkaline Proteases

The highest alkaline protease activity was recorded within bees exposed to an E-field with an intensity of 5.0 kV/m while the lowest was in the control bees. Among the experimental groups, the least influence on alkaline protease activity was for an intensity of 23.0 kV/m (Figure 1). The control group and the 23.0 kV/m group did not differ significantly (Table 1). Changes in the activity of alkaline proteases between the experimental groups and the control group were smaller compared to acidic and neutral protease activity (*p*-value < 2.2 × 10^−16^).

### 3.5. Behavior Analysis

**Figure 2:**
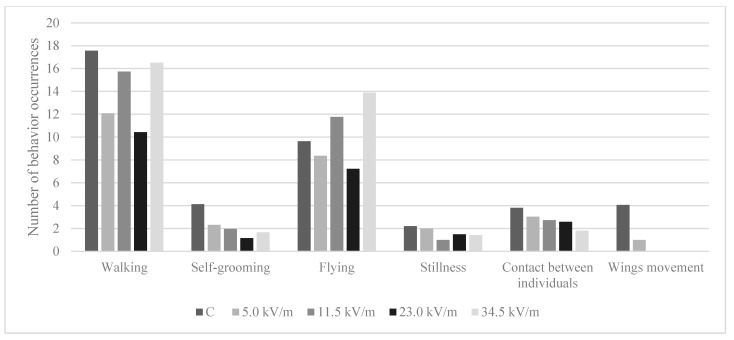
The average number of behavior occurrences displayed by bees after 12 h under the influence of an E-field at 50 Hz and an intensity of 5.0 kV/m, 11.5 kV/m, 23.0 kV/m, or 34.5 kV/m. The group name is the E-field intensity to which the bees were exposed. The control group is marked with the letter C.

**Figure 3:**
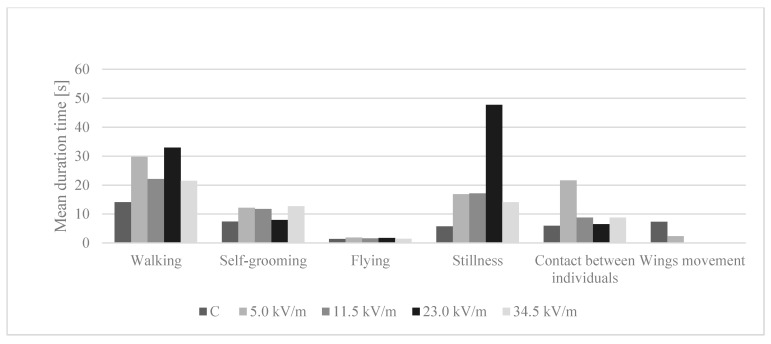
Average time spent on behavior by bees after 12 h under the influence of an E-field at 50 Hz and an intensity of 5.0 kV/m, 11.5 kV/m, 23.0 kV/m, or 34.5 kV/m. The group name is the E-field intensity to which the bees were exposed. The control group is marked with the letter C.

Bees in the control group and the 5.0 kV/m group displayed all six behaviors (Figure 2 and Figure 3). For the 11.5 kV/m, 23.0 kV/m, and 34.5 kV/m groups, all behaviors were observed except wing movement. The number of stillness and wing movement observations was too small to show statistically significant differences between the groups (Table 2 and Table 3).

#### 3.5.1. Number of Behavioral Occurrences

The most frequently shown behavior in all groups was walking and the least stillness or, in the case of the 5.0 kV/m group, wing movement, which was noticed only once (Figure 2, Table 2). Bees in the control group demonstrated the highest number of all behavior occurrences, except flying, which was most often displayed by the 34.5 kV/m group (*p*-value = 0.0001). Differences in walking occurrences between the control group and all experimental groups were statistically significant (Table 2) (*p*-value < 6.96 × 10^−7^). The 23.0 kV/m group displayed the lowest number of flying, walking, and self-grooming occurrences. Contact between individuals was displayed least frequently by the 34.5 kV/m group and stillness by the 11.5 kV/m group. The number of occurrences of contact between individuals decreased with increasing intensity; however, a statistically significant difference occurred only between the control group and the 34.5 kV/m group (Figure 2, Table 2) (*p*-value = 0.0032).

#### 3.5.2. Time Spent Performing Behaviors

Bees in the control group, the 5.0 kV/m, 11.5 kV/m, and 34.5 kV/m groups, on average, spent most of the time walking (Figure 3). The average time spent walking by the control group statistically differed from the other groups (Table 3) (*p*-value = 3.69 × 10^−8^). In the 23.0 kV/m group, the bees remained still most of the time. In all groups, the bees spent the least time flying (*p*-value = 0.0001). The control group displayed the shortest average duration of all behaviors, except wing movement. Walking was displayed the longest by bees in the 23.0 kV/m group, self-grooming by bees in the 34.5 kV/m group, flying by bees in the 5.0 kV/m group, stillness by bees in the 23.0 kV/m group, and contact between individuals in the 5.0 kV/m group.

## 4. Discussion

### 4.1. Protease Analysis

Proteases are enzymes that catalyze the hydrolysis of peptide bonds which link amino acid residues [30]. They occur in all types of organisms—eucaryotic, procaryotic, and viruses [31]. Proteases are involved in physiological reactions, e.g., digestive, apoptosis, blood clotting. The material of our research was the hemolymph in which serine proteases occur [32]. This group of proteases plays an important role in regulatory and signaling processes, digestion, transport, and degradation of the damaged protein. Serine proteases are involved in the correct action of insect resistance barriers and antioxidant systems. They are responsible, among others functions, for melanization, wound healing, and phagocytosis stimulation by taking part in phenol pro-oxidase cascade activation [33]. Based on the optimal pH in which these enzymes are active, proteases can be classified as alkaline (basic), neutral, or acid.

In our present study, treating bees with an E-field at 50 Hz and an intensity of 5.0, 11.5, 23.0, or 34.5 kV/m for 12 h caused an increase in the level of proteases in comparison to the control group (Figure 1, Table 1). The activity of proteases did not increase with increasing electromagnetic field intensity. In our previous study [13], bees exposed to an E-field with identical parameters for 1, 3, and 6 h had higher acid and neutral protease activity than the control group. Regarding alkaline proteases, their activity was higher only in those bees treated with an E-field with an intensity of 23.0 or 34.5 kV/m. Changes in the protease activity in the honey bee organism after long exposure (12 h) to an E-field still persist. The increased activity of proteases in hemolymph was also noticed after treating bees with bromfenvinphos [34]. The authors assumed that *Varroa* treatment with bromfenvinphos markedly suppresses the honey bee biochemical defense levels. We hypothesized that increased protease activity after E-field exposure could also cause such effects.

Changes in protease activity occurred after treating bees with curcumin, coenzyme Q10, or caffeine. The addition of curcumin and coenzyme Q10 in sugar syrup caused the decreased activity of proteases in hemolymph [35,36]. Caffeine caused an increase of neutral protease activity and a decrease of alkaline and acidic protease activity [37]. Bees treated with Q10, curcumin and caffeine lived longer than control bees [35,36,37]. Based on these studies, it can be assumed that these substances have a potentially positive effect on longevity by decreasing protease activity. Strachecka et al. [15] assumed that the activity of proteases on the body surface differs in polluted and clean environments.

Additionally, after treating bees with imidacloprid, a decrease in acidic and alkaline protease activity and an increase in neutral protease activity was noticed regardless of the doses of insecticide (5 or 200 ppb) [38].

It is difficult to clearly state what effect the increased activity of proteases has on bees’ immunity. Decreases and increases of protease activity occur in healthy bees and are connected with the age of the insect. The increase of acidic, neutral, and alkaline protease activity can be noticed until the age of 18–20 days and decreases after this time [34].

### 4.2. Behavior Analysis

Behavior plays a significant role in insect immunity. Bees as social insects have evolved mechanisms of individual and social behavioral defense that can minimalize the presence of pathogens, pests, and parasites [17,18]. Factors that threaten bees often affect insect behavior and change their activity. These phenomena can influence disease susceptibility by affecting behaviors related to immune responses, like self-grooming (even when the threatening factor does not alter the immune response of the bee).

In our study, treating bees with an E-field at 50 Hz and an intensity of 5.0, 11.5, 23.0, or 34.5 kV/m for 12 h caused a reduction in the number of occurrences self-grooming, contact between individuals, and walking while increasing the average time spent on the behavior in comparison to the control group (Figure 2 and Figure 3). Contact between individuals is a significant behavior in pheromone transmission and any disorder of this behavior results in a modification of the relationships between individuals and thus community functioning. Self-grooming is an important trait that contributes to the defense against pests, pathogens, and parasites. Thus, if an E-field changes the behavioral pattern of the honey bee, it can indirectly affect the honey bee’s immune system.

Since bees in the control group changed their behavior more often than the experimental bees, the average time spent on an individual behavior was shorter in the control group (Figure 2 and Figure 3). Bees in the experimental groups were less active (changing their behavior less often). Our previous study shows that treating bees with an E-field with the same parameters for 1, 3, and 6 h caused a similar behavioral change. Bees in the experimental group were cleaning themself and displayed contact between individuals less frequently than the control bees [9]. Our present study shows that bees after longer exposure (12 h) to an E-field still displayed modified behavioral patterns compared to the control bees.

Only a few publications have evaluated the impact of an E-field on honey bee behavior; thus, a comparison of the results to other factors was necessary. Changes in honey bee activity were observed in studies on the effect of pesticides on bees, in particular, neurotoxins, which affect neural processes by affecting the conduction of the electrical signals between nerve cells (neurons). We can hypothesize that an E-field can also change the processing and conduction of nerve impulses. Depending on the dose, imidacloprid may induce an increase or a decrease in activity. The lowest dose used in the studies by Lambin et al. [39] (1.25 ng per bee) caused an increase in motor activity, while higher doses (2.5–20 ng per bee) caused a decrease. In sublethal doses, cypermethrin, tetramethrin, and tau-fluvalinate reduced the motor activity in honey bees. In our present study, the E-field caused a reduction in the number of occurrences of most behaviors (Table 2, Figure 2). Control bees were more active—they changed behavior more often (41 times on average during the whole observation time) than bees in the experimental groups (from 23 to 35 times). James and Xu [40] assumed that by influencing the motor activity of bees, neurotoxic insecticides can affect disease resistance even not affecting individual immunity.

Imidacloprid provoked problems with coordination, convulsions, excessive agitation, or stillness in honey bees [39,41,42]. Exposure for 12 h to an E-field caused stillness to be displayed by the bees, but the number of occurrences of this behavior was too small to show significant differences (Table 2 and Table 3). Even so, it is worth paying attention to the fact that the control bees were still for 5.72 s, while bees in the experimental groups were motionless for from 14.07 s (34.5 kV/m group) to 47.71 s (23.0 kV/m group) (Table 3). Bee stillness after contact with imidacloprid results from the disturbance of impulse conduction caused by a binding of this insecticide with acetylcholine receptors [39]. Morfin et al. [43] found that chronic sublethal exposure to clothianidin affected the proportion of bees grooming intensively and, based on RNAseq, found an effect on pathways linked to neural function, which could be related to the bees’ ability to perceive external stimuli. It is possible that E-field and neurotoxic insecticides can cause changes in the bee nerve impulse transmission; however, the mode of action is probably different. Bees exposed to esfenvalerate and permethrin spent, respectively, 43% and 67% less time in social interaction compared to control bees [44]. Bees in our present work treated with an E-field with an intensity of 34.5 kV/m or 11.5 kV/m spent on average 47% less time in contact between individuals than control bees, while bees in the 23.0 kV/m group spent on average 8% less time on this behavior. Bees treated with an E-field with an intensity of 5.0 kV/m spent on average 262% more time in contact between individuals. Nevertheless, bees in the control group displayed this behavior more often than the other groups (Figure 2 and Figure 3; Table 2 and Table 3).

Chronic exposure to thiamethoxam significantly impairs a bee’s ability to fly: It reduces the flight time (−54%), flight distance (−56%), and its average speed (−7%) [45]. According to our present study, bees in the control group spent the least time flying, although, the differences between the groups, except the 5.0 kV/m group, were not statistically significant (Figure 3, Table 3). Regarding the number of flying occurrences, the 34.5 kV/m group had the highest number of this behavior occurrence (Figure 2). The difference between this group, the control group, and the 23.0 kV/m group was not statistically significant. The bees in the 5.0 kV/m and 11.5 kV/m group flew significantly fewer times than bees in the 34.5 kV/m group (Table 2). In conclusion, E-fields cause changes in bee behavior, most often by reducing the number of occurrences of individual behavior.

### 4.3. Behavior and Protease Analysis Comparison

In our present study, the level of protease activity increased in the experimental bee organisms (Figure 1). Experimental bees also displayed a reduced number of behavior occurrences in comparison to control bees (Figure 2). In our previous study, bees that were treated with an E-field for 1, 3, or 6 h have higher activity of neutral and acidic proteases than control bees. In the case of alkaline proteases, only bees treated with intensities of 23.0 or 34.5 kV/m have a statistically significant higher activity of this enzyme than control bees [13]. Our previous behavioral research shows that bees in the experimental groups have a reduced number of contacts between individuals and self-grooming occurrences [9].

Bees’ immune system response consists both of individual and social immunity, which includes anatomical barriers, cellular and humoral immunity, and behavioral immunity [17,18]. Serine proteases which occur in hemolymph are responsible for melanization, wound healing, and phagocytosis stimulation [33].

Based on our present and previous studies [9,13], it can be concluded that bees after E-field exposure are characterized by higher protease activity and reduced contact with other individuals and clean themself less often. Protease activity and behavior parameters analysis can give valuable information about the effect of an E-field on the bees’ immunity. Changes in these parameters may indicate the interaction of behavioral immunity and protease activity, which are designed to protect honey bee’s organisms against environmental stressors (pesticides, pathogens, etc.)

## 5. Conclusions

The amount of artificial electromagnetic field in the environment is constantly increasing, thus the honey bee is exposed to this factor. In our study, bees in the control group demonstrated the highest number of all behavior occurrences, except flying, and had the lowest activity of all types of proteases. Bees in the experimental groups showed a lower number of walking, self-grooming, and contact between individual occurrences than control bees and had higher protease activity than control bees. Our results show that an E-field is potential harmful factor to the honey bee. However, we do not know if the changes in behavior and protease activity of the honey bee after E-field exposure persist and for how long. It would be important to investigate behavior parameters and biochemical markers at different time intervals after the end of exposure to an E-field. It can be helpful to determine the interaction between the biochemical marker activity and behavioral parameters. Such an observation could provide a better understanding of the immune response of the honey bee exposing to environmental stressors.

## Figures and Tables

**Table 1:** Comparison of protease activity between all groups.

Groups	Protease Activity (U/mg Protein)		
	Acidic	Neutral	Alkaline
C	1.18 (±0.11) ^a^	2.45 (±0.07) ^a^	1.35 (±0.10) ^a^
5.0 kV/m	2.09 (±0.24) ^b^	4.26 (±0.26) ^b^	1.89 (±0.25) ^b^
11.5 kV/m	1.92 (±0.08) ^c^	3.94 (±0.08) ^c^	1.42 (±0.11) ^c^
23.0 kV/m	2.84 (±0.07) ^d^	5.52 (±0.25) ^d^	1.40 (±0.09) ^ac^
34.5 kV/m	4.24 (±0.32) ^e^	4.26(±0.11) ^b^	1.72 (0.20) ^d^

The average protease activity in bee organisms after 12 h under the influence of an E-field at 50 Hz and an intensity of 5.0 kV/m, 11.5 kV/m, 23.0 kV/m, or 34.5 kV/m. The group name is the E-field intensity to which the bees were exposed. The control group is marked with the letter C. Standard deviation is shown in round brackets. Different letters (a, b, c, d, e) indicate statistical differences between the groups at the *p* ≤ 0.05 significance level.

**Table 2:** Comparison of the number of behavior occurrences between all groups.

Groups	Behavior Parameters					
	Walking	Self-Grooming	Flying	Stillness	Contact between Individuals	Wing Movement
C	17.57 (±5.83) ^a^	4.13 (±2.35) ^a^	9.64 (±6.89) ^ab^	2.22 (±1.92)	3.82 (±2.70) ^a^	4.07 (±3.07)
5.0 kV/m	12.08 (±9.05) ^b^	2.33 (±1.88) ^ab^	8.37 (±6.29) ^a^	2.00 (±1.41)	3.03 (±2.38) ^ab^	1.00
11.5 kV/m	15.74 (±10.32) ^bc^	1.97 (±1.97) ^b^	11.77 (±9.57) ^a^	1.00 (±0.01)	2.73 (±1.41) ^ab^	NO
23.0 kV/m	10.44 (±6.45) ^b^	1.17 (±0.39) ^b^	7.23 (±6.05) ^ab^	1.50 (±0.71)	2.59 (±1.04) ^ab^	NO
34.5 kV/m	16.51 (±9.28) ^c^	1.68 (±1.07) ^b^	13.90 (±9.53) ^b^	1.44 (±0.73)	1.81 (±0.88) ^b^	NO

The average number of the behavior occurrences displayed by bees after 12 h under the influence of an E-field at 50 Hz and an intensity of 5.0 kV/m, 11.5 kV/m, 23.0 kV/m, or 34.5 kV/m. The group name is the E-field intensity to which the bees were exposed. The control group is marked with the letter C. Standard deviation is shown in round brackets. Different letters (a, b, c) indicate statistical differences at the *p* ≤ 0.05 significance level. NO—not observed.

**Table 3:** Comparison of the average time spent on each behavior between the groups (s).

Groups	Behavior Parameters					
	Walking	Self-Grooming	Flying	Stillness	Contact between Individuals	Wing Movement
C	14.07 (±6.47) ^a^	7.43 (±8.87) ^a^	1.38 (±0.45) ^a^	5.72 (±2.86)	5.99 (±4.00) ^a^	7.36 (±6.07)
5.0 kV/m	29.78 (±22.95) ^bc^	12.18 (±11.82) ^b^	1.86 (±0.52) ^b^	16.87 (±15.78)	21.66 (±20.89) ^b^	2.40
11.5 kV/m	22.09 (±14.20) ^b^	11.78 (±10.70) ^b^	1.60 (±0.46) ^ab^	17.15 (±20.43)	8.80 (±7.93) ^c^	NO
23.0 kV/m	32.94 (±16.75) ^c^	7.98 (±6.80) ^a^	1.73 (±0.72) ^ab^	47.71 (±12.52)	6.48 (±3.73) ^ac^	NO
34.5 kV/m	21.46 (±13.41) ^b^	12.75 (±10.43) ^b^	1.45 (±0.25) ^a^	14.07 (±13.54)	8.78 (±7.19) ^c^	NO

Average time (s) spent on each behavior by bees after 12 h under the influence of an E-field at 50 Hz and an intensity of 5.0 kV/m, 11.5 kV/m, 23.0 kV/m, or 34.5 kV/m. The group name is the E-field intensity to which the bees were exposed. The control group is marked with the letter C. Standard deviation is shown in round brackets. Different letters (a, b, c) indicate statistical differences at the *p* ≤ 0.05 significance level. NO—not observed.

## Data Availability

The datasets generated during and/or analyzed during the current study are available from the corresponding author on reasonable request.
